# Clinical and Pathologic Profiles of Esophageal Cancer in Mozambique: A Study of Consecutive Patients Admitted to Maputo Central Hospital

**DOI:** 10.1200/JGO.18.00147

**Published:** 2018-11-06

**Authors:** Jotamo Come, Clara Castro, Atílio Morais, Matchecane Cossa, Prassad Modcoicar, Satish Tulsidâs, Lina Cunha, Vitória Lobo, Alberto Gudo Morais, Sofia Cotton, Nuno Lunet, Carla Carrilho, Lúcio Lara Santos

**Affiliations:** **Jotamo Come**, **Atílio Morais**, **Matchecane Cossa**, **Prassad Modcoicar**, **Satish Tulsidás**, **Lina Cunha**, **Vitória Lobo**, **Alberto Gudo Morais**, and **Carla Carrilho**, Hospital Central de Maputo; **Carla Carrilho**, Universidade Eduardo Mondlane, Maputo, Moçambique; **Clara Castro**, **Sofia Cotton**, and **Lúcio Lara Santos**, Instituto Português de Oncologia; **Clara Castro** and **Nuno Lunet**, Universidade do Porto; **Sofia Cotton**, Project ESTIMA-01-0145-FEDER-000027; **Sofia Cotton** and **Lúcio Lara Santos**, Grupo de Patologia e Terapêutica Experimental; **Lúcio Lara Santos** Universidade Fernando Pessoa, Porto, Portugal.

## Abstract

**Purpose:**

Eastern Africa was recently described as a high-incidence geographic area for esophageal cancer. Mozambique is included in this region. This study aimed to characterize this malignant disease at Maputo Central Hospital (MCH) to develop a global program for esophageal cancer management in Mozambique.

**Methods:**

MCH records from between 2012 and 2016 were used to assess the clinical, pathologic, and outcome profiles of esophageal tumors. A descriptive analysis of data collected was performed. Overall survival was evaluated using Kaplan-Meier curves.

**Results:**

In the study, 522 consecutive patient cases of esophageal cancer were recorded. The median patient age was 56.1 years (range, 27 to 97 years); 291 (55.7%) patients were women, and 230 (44.1%) were men. Regarding tumor site, 113 patients (21.6%) had a tumor in the lower third, 154 (29.5%) in the middle, and 50 (9.6%) in the upper third of the esophagus; in the remaining 196 (37.5%), tumor site was unknown. Squamous cell carcinoma comprised 94.4% of cases with documented histopathology (74.9% of the sample). Surgical treatment was possible in 32 patients (6.1%). Disease stage was documented only in these 32 surgical patients; 28.1%, 53.1%, and 18.8% had stage I, II, and III disease, respectively. The remaining patient cases seemed to involve clinically advanced tumors. The median follow-up time was of 1.6 months. The median survival time was of 3.5 months for all patients; for patients treated with curative intent, it was of 8.7 months.

**Conclusion:**

Esophageal carcinoma is a common malignant tumor at MCH and is diagnosed in the advanced stages resulting in poor prognosis. Therefore, implementation of an Esophageal Cancer Program in Mozambique is essential.

## INTRODUCTION

Esophageal cancer is the eighth most frequent cancer worldwide and sixth most common cause of death resulting from cancer.^1^ The distribution of esophageal cancer varies geographically, with 80% of cases occurring in developing countries.^2^ The highest incidences of esophageal cancer in the world have been observed in China, northeastern Iran, southeastern United States, and Southern Africa.^3^ Among both sexes, there are more than 20-fold differences in incidence in world regions, with rates ranging from 0.8 per 100,000 in West Africa to 17.0 per 100,000 in East Asia in men and 0.2 per 100,000 in Micronesia/Polynesia to 7.8 per 100,000 in East Africa in women.^4^ Mortality rates are elevated in East Asia (14.1 per 100,000) and Southern Africa (12.8 per 100,000) in men and in East Africa (7.3 per 100,000) and Southern Africa (6.2 per 100,000) in women.^4^ The high incidence rates of esophageal cancer (esophageal squamous cell carcinoma) in Central, Southern, and East Africa, together with diagnosis at advanced stages, lead to high mortality rates.^1,2,5-7^ The reasons underlying the high frequency of this malignancy on that continent remain largely unknown, and investigation to evaluate potential etiologic effects of dietary, lifestyle, environmental, and other factors affecting incidence in this region, including genetics, is needed.^2,8-10^ In their study, Liu et al^11^ demonstrated discrete subtypes of esophageal squamous cell carcinoma in sub-Saharan Africa and suggested that the endemic nature of this disease reflects exposure to a carcinogen other than tobacco or oncogenic viruses.

In Mozambique, data on the basis of the Maputo Central Hospital (MCH) registry reveal that esophageal cancer is the fourth most frequent tumor for both sexes, and it is the most frequent occurring in the digestive tract.^12,13^ Dysphagia related to esophageal cancer has been a major cause of hospitalization. Many of these patients are admitted with severe malnutrition and dehydration resulting from difficulties in swallowing, which are related to poor prognosis.^14^ With the aims of creating a global program to fight this disease, promoting good practices, and empowering disease management, MCH records were used to evaluate the burden of esophageal cancer in the hospital; assess the clinical and pathologic profiles of such tumors, treatment approach; and barriers in collection of data in this context; and define a plan of action that can contribute to the improvement of care management for these patients. The study was performed at MCH, located in Maputo, the capital of Mozambique. It is a 1,500-bed quaternary hospital and serves the Eduardo Mondlane University Medical School as a teaching hospital. MCH is the national reference hospital of the country.

## METHODS

MCH records were retrospectively assessed from the Pathology and Surgery Services database to obtain the clinical and pathologic characteristics of patients with esophageal cancer admitted to and treated at MCH between January 1, 2012, and April 30, 2014. From May 2014 to December 2016, data were obtained from the MCH hospital-based cancer registry. This registry was implemented in May 2014 and includes information about patient cases of esophageal cancer identified in the departments of pathology and oncology, as well as data on cancer-related deaths from the Intrahospital Death Registration System.^13^ In addition, surgery department files were also assessed to complete information on patients diagnosed on the basis of clinical data only. Demographic variables collected for each patient included sex, age, race, and origin of the patient; clinical and pathologic data included anatomic site, histologic subtype, grade, basis of diagnosis, and treatments performed for each patient (surgery and chemotherapy). For patients undergoing surgery with curative intent, information on stage (American Joint Committee on Cancer seventh edition^15^), tumor size, resection status, and occurrence of lymphatic, vascular, muscular, perineural, adventitia, and/or lymph node invasion was also collected. A descriptive analysis of data collected was performed to describe the clinicopathologic profile of esophageal cancer at MCH. Overall survival was evaluated using Kaplan-Meier curves. The study was approved by the Mozambican National Bioethics Committee (reference No. 432/CNBS/2017).

## RESULTS

**Fig 1:**
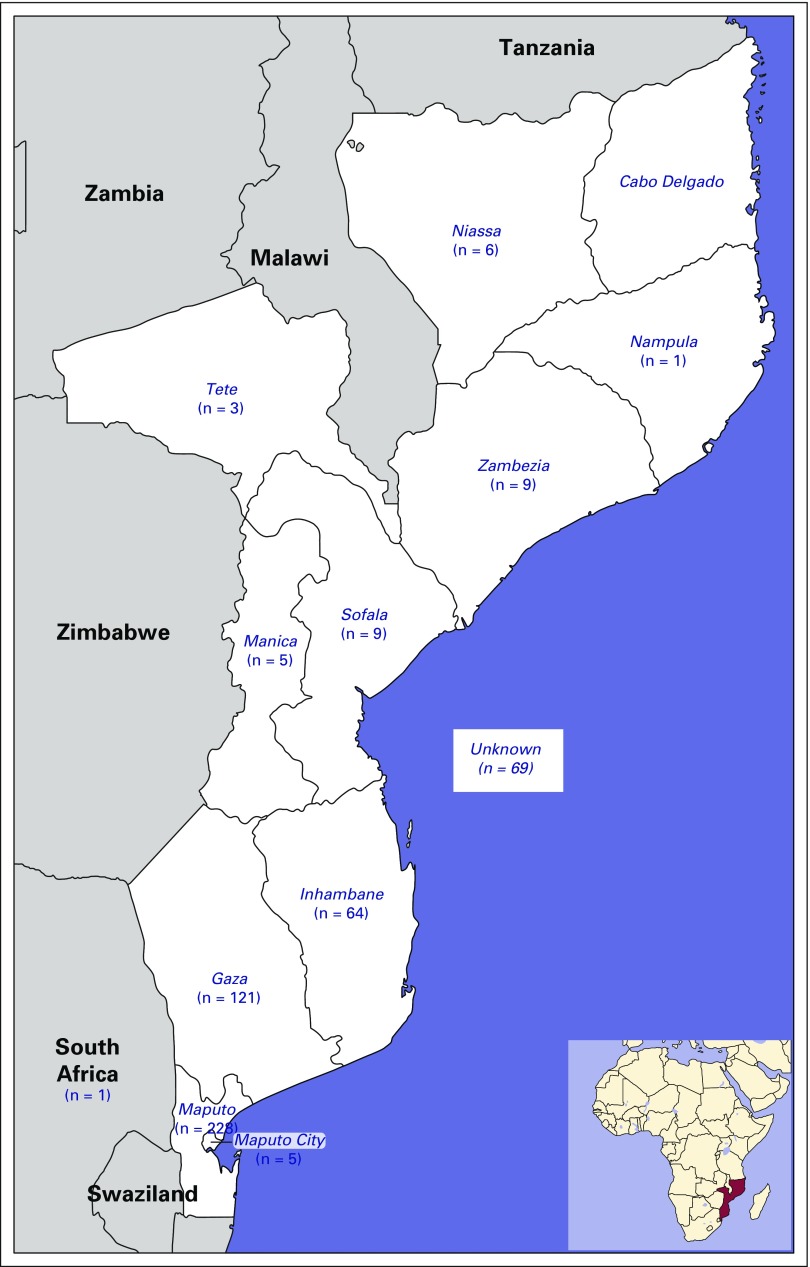
Geographic distribution of place of birth of patients by provinces in the country.

**Fig 2:**
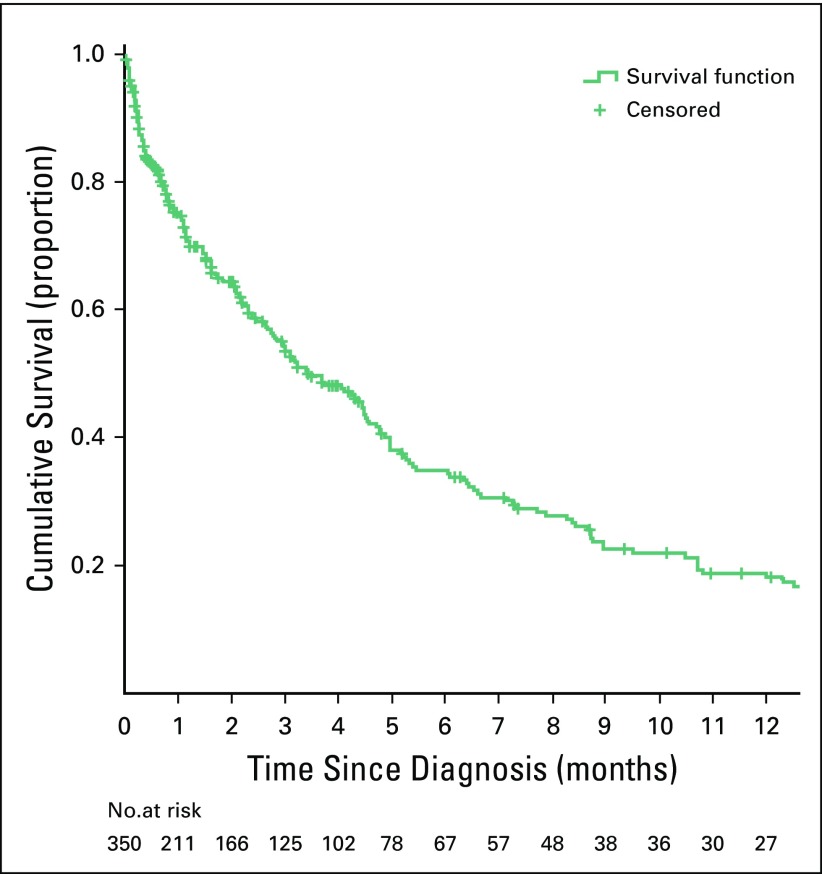
Overall survival of patients with esophageal cancer since date of diagnosis.

Over the study period, 522 consecutive patient cases of esophageal cancer were recorded (Table 1). The mean age at diagnosis was 56.1 years (standard deviation, 13.23 years; range, 23 to 97 years); 53 patients (10.2%) were age < 40 years; 14 patients (2.6%) were age ≤ 30 years. Most patients were women (n = 291; 55.7%), black (n = 520; 99.6%), and born in southern regions of the country (n = 418; 80.1%; Fig 1). The male-to-female ratio was between 0.7 and 0.9 in all 10-year age groups (20 to 29, 30 to 39, 40 to 49, 50 to 59, 60 to 69, and ≥ 70 years). Dysphagia to solids was documented in all patients and required nutritional support. However, nutritional management with gastrostomy is unsuccessful in a large number of patients. Information on the anatomic site of the tumor was not available for 196 patients (37.5%); among the remaining patients, 154 (47.2%) had a tumor located in the middle third of the esophagus, 113 (34.7%) in the lower third, 50 (15.3%) in the upper third, and nine (2.8%) in the esophagogastric junction (Table 1). Diagnosis was histologically confirmed for 391 patient cases (74.9%; Table 1). Among those, most cases were squamous cell carcinomas (n = 369; 94.4%); 10 (2.6%) were adenocarcinomas, and three (0.8%) were adenosquamous carcinomas. Among the 192 patient cases with available information on histologic grade, 83 (43.2%) were well differentiated, 88 (45.8%) were moderately differentiated, and 21 (10.9%) were poorly differentiated. In the studied series, 55.8% of patients underwent no staging imaging. Surgical treatment with curative intent (Ivor-Lewis procedure) was only possible in 32 patients (6.1%); surgical feeding gastrostomy was performed using a urinary catheter tube in 199 patients (38.1%), and the remaining 291 patients (55.7%) received best support care. For patients undergoing surgery with curative intent, the median tumor size was 40 mm (interquartile range, 25.5 mm; range, 15 to 100 mm); 28.1%, 53.1%, and 18.8% of patients had stage I, II, and III disease, respectively; 21 patients (65.6%) had negative margins; and lymphatic, vascular, perineural, muscular propria, adventitia, and lymph node invasion were found in 14 (43.8%), 14 (43.8%), 11 (34.4%), 31 (96.9%), 20 (64.5%), and eight patients (25.0%), respectively. The neoadjuvant chemotherapy protocol used (cisplatin 75-100 mg/m^2^ intravenously on day 1 plus fluorouracil 1,000 mg/m^2^ continuous infusion over 24 hours on days 1 to 5. Cycled every 28 days), included four cycles before operation and two after the operation. In adjuvant/palliative context the same protocol was used during six cycles. Chemotherapy was administered to 18 patients (3.4%).Only one patient received neoadjuvant chemotherapy; for the remaining 17 patients, chemotherapy was adjuvant or palliative. Radiotherapy was not available. In survival analyses, 172 patients (32.9%) were excluded (127 patients were lost to follow-up, 26 had as date of diagnosis the date of death, and 19 had only the year of diagnosis and/or follow-up available.). The median follow-up time was of 1.6 months (1.3 *v* 2.1 months for alive and dead patients, respectively, at the date of last contact). The median survival time was of 3.5 months for all patients (Fig 2), whereas for patients treated with curative intent (either surgery alone or with neoadjuvant chemotherapy, according to stage), it was of 8.7 months.

**Table 1:**
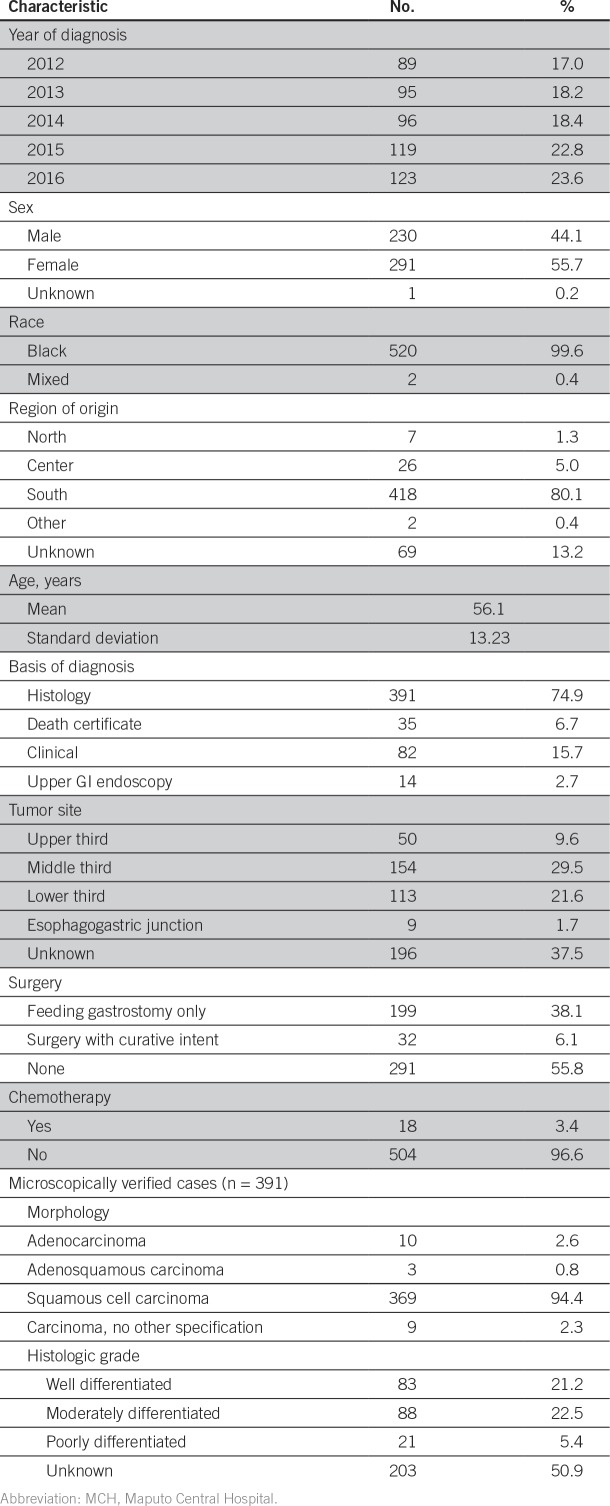
Descriptive Analysis of Demographic and Clinical Characteristics of Patients With Esophageal Cancer Registered at MCH Over Study Period (N = 522)

## DISCUSSION

This study reveals that esophageal cancer is a common condition at MCH. The surgery, gastroenterology, medical oncology, and pathology departments in the hospital were the sources of information used in this study. However, a multidisciplinary approach toward treating esophageal cancer was nonexistent. In our study sample, most patients were black women. The sex ratio found in our study was different from that in other African countries; however, it was closer to values found in Ethiopia, Kenya, and Sudan; furthermore, a decrease in sex ratio to increasing incidence of women (used as an indicator of underlying incidence in the general population) was described in a recent report.^16^ In the same study, a sex ratio of 1.4 was reported by another cancer registry in Mozambique (Beira Cancer Registry), but only 30 patients were included in the analyses, hindering extrapolation to the general population in that country. Although no information on migration or place of residence could be obtained for the patients included in our study, a report related to the study of migration in Mozambique reveals that there are low internal migration rates in Mozambique, so this lack of information is not expected to have greatly influenced our results.^17^ Diagnosis was usually determined at an advanced stage of disease, precluding the curative approach, and nutritional status at diagnosis was usually poor according to clinical data. In a South African study involving 1,868 patients, similar stage, performance status, weight loss, and long history of dysphagia were described; furthermore, only 19.8% of those patients could be treated with curative intent, and squamous cell carcinoma was also the most frequent histologic type.^7^ In our study, most patients required nutritional support, as observed in South African reports.^7,18^ Gastrostomy was the most frequently performed palliative surgery, but it is often responsible for complex skin lesions and additional suffering.^19,20^ Stent palliation represents a more efficient approach.^21^ However, the retail price for the imported stent is a barrier.^22^ Therefore, we have performed palliative surgical procedures, such as bypass. The clinical and pathologic profiles observed in our study are similar to those observed in other African countries in the region.^21-23^ This study shows that it is necessary to implement a global esophageal cancer program in Mozambique and at MCH, aiming to increase the imaging stage and treatment of esophageal cancer with radical surgery, chemoradiotherapy, neoadjuvant and palliative chemotherapy, and endoscopic palliation of dysphagia (Table 2). Soon, the use of radiotherapy will also be available in this hospital, which, together with the nutrition and clinical psychology departments, will have an important role in cancer treatment and prehabilitation of surgical patients.^24^ The African Esophageal Cancer Consortium, the call to action in esophageal cancer by von Loon et al,^22^ could be a useful platform to implement multisite investigation in this field and capacity building and to share research in treatment and palliative care, including the palliation of dysphagia. However, risk factors associated with esophageal carcinogenesis in Mozambique and East Africa are largely unknown.^25^ In sub-Saharan Africa, case-control studies have shown that low economic level, consumption of local fermented beverages and foods cooked with charcoal, and smoking may be associated with a high risk of esophageal cancer.^8,24-29^ Deficits of selenium and zinc in foods also seem to have an important role in malignant transformation of esophageal epithelium in this region.^30^ In Kenya, endoscopic evaluation of asymptomatic individuals living in regions with high esophageal cancer rates revealed that 14.4% of patients had dysplastic lesions.^31^ In this study, the identification of risk areas was performed using Lugol’s solution. However, endoscopy using narrow band imaging seems more effective for those with an early diagnosis of esophageal cancer.^32^ In Mozambique, there is currently no information on these issues; therefore, it is necessary to study the local risk factors and promote early diagnosis in high-risk areas (in which the provinces of Gaza, Inhambane, and Maputo seem to be included according to the present data), possibly using endoscopy through narrow band imaging, because this technical resource is available at the MCH gastroenterology department, with Lugol staining improving diagnosis. However, large tumors that prevent endoscopic stent placement, as well as the difficulty of managing the reported feeding gastrostomies, suggest that the role of palliative surgery associated with chemotherapy or radiotherapy should be evaluated in a scientific way in this context.^33^

**Table 2:**
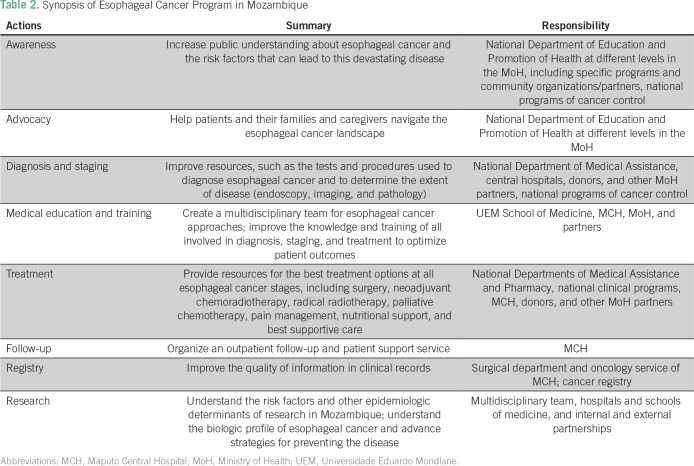
Synopsis of Esophageal Cancer Program in Mozambique

This study has several limitations that need to be addressed. For 25% of our patients, the diagnosis was on the basis of clinical diagnosis only, endoscopy, or death certificate. The data obtained are from those patients for whom clinical records were available. As a retrospective study, there is a lack of information on some variables (eg, staging, time of follow-up, and outcomes for some patients). In order to overcome these limitations, MCH is currently implementing a hospital-based cancer registry comprising data from 2014 onward that integrates relevant sources of information. ^13^ However, the quality of information produced by the registry is dependent not only on the organization and availability of the hospital records but also importantly on the quality of the information included in such records. For example, information about disease stage was not present in almost all clinical records and was only available for patients undergoing surgery with curative intent, when this information referred to pathologic stage. A recent study conducted in Tanzania^34^ reported a clinical profile and problems similar to those described in our study, which reinforces the need for a regional intervention in this disease.

Esophageal cancer is a commonly diagnosed and treated condition at MCH, but diagnosis is made at an advanced stage of disease, which is related to poor prognosis. This study reveals the necessity for the development of a comprehensive program to combat esophageal cancer in Mozambique.
